# Maryland University Dental Department Building

**Published:** 1882-07

**Authors:** 


*Maryland University Dental Department Building.*—By the time the present Number of the JOURNAL is issued the extensive Dental Infirmary and Laboratory Building of the Dental Department of the University of Maryland will be completed and fully equipped with complete dental appliances. This is the only structure, so far as we know, that has ever been built solely for dental use, and hence its arrangements have been most carefully attended to with a view of having it meet all the requirements of a building devoted to infirmary and laboratory uses. Although the Infirmary Hall, which occupies all of the second story is not so large as that of the University of Pennsylvania, Dental Department, it excells all others in size, and by its position affords unobstructed light by means of twenty-five large windows, a convenience which no other Dental Infirmary, not even the one just referred to can claim. The first floor contains the extensive Laboratory (the largest we believe in existence,) the Extracting Room and the Impression Room.

The Laboratory will accomodate, as at present arranged, one hundred students with separate desks and work benches for mechanical work of every description, besides containing all of the most recent and improved apparatus, rendering it superior to any private laboratory whatever.

Marble top wash stands, water closet, closet for students instruments, and a cloak room complete the arrangements of this large and superior dental structure, which will afford every facility for practical instruction in both operative and mechanical dentistry.

The Operating Hall is supplied with Morrison and Archer chairs, one before each window, the majority being of the former style, with a large number of dental engines, brackets for instruments, electric mallet and battery and gas apparatus, etc., etc. The Infirmary and Laboratory are both capable of accomodating several hundred students.

